# A Review of Durability and Strength Characteristics of Alkali-Activated Slag Concrete

**DOI:** 10.3390/ma12081198

**Published:** 2019-04-12

**Authors:** Osama Ahmed Mohamed

**Affiliations:** College of Engineering, Abu Dhabi University, P.O. Box 59911, Abu Dhabi, UAE; osama.mohamed@adu.ac.ae; Tel.: +971-02-501-5752

**Keywords:** alkali activated slag, carbonation, shrinkage, alkaline activator, hydration products, alkali-silica reaction, curing temperature, fly ash, silica fume

## Abstract

Alkali-activated slag (AAS) is a promising alternative to ordinary Portland cement (OPC) as sole binder for reinforced concrete structures. OPC is reportedly responsible for over 5% of the global CO_2_ emission. In addition, slag is an industrial by-product that must be land-filled if not re-used. Therefore, it has been studied by many investigators as environmentally friendly replacement of OPC. In addition to recycling, AAS offers favorable properties to concrete such as rapid development of compressive strength and high resistance to sulfate attack. Some of the potential shortcomings of AAS include high shrinkage, short setting time, and high rate of carbonation. Using ground granulated blast furnace slag (GGBS) as an alternative to OPC requires its activation with high alkalinity compounds such as sodium hydroxide (NaOH), sodium sulfate (Na_2_SO_3_), sodium carbonate (Na_2_CO_3_), or combination of these compounds such as NaOH and Na_2_SO_3_. The mechanism of alkali-activation is still not fully understood and further research is required. This paper overviews the properties, advantages, and potential shortcomings of AAS concrete.

## 1. Introduction

The replacement of cement with alternative industrial by-products characterized with high percentages of alumina and silica (aluminusilicates) activated with alkalis has been researched for decades, but is gaining popularity at the present time due to increased interest in reducing environmental footprint of cement production. Aluminusilicates, the most commonly used source materials as alternatives to ordinary Portland cement (OPC), contain relatively large amounts of silicon oxide (SiO_2_) and aluminum oxide (Al_2_O_3_) [1]. Despite certain shortcomings in comparison to OPC concrete, such as high carbonation and shrinkage, AAS concrete offers many opportunities to the construction industry in terms of durability and high early strength development. The high early strength development in particular makes AAS a viable alternative to OPC for deep-water oil well cementing [2].

Anhydrous GGBS (ground granulated blast furnace slag) contains higher amounts of SiO_2_ compared to OPC (30.04–35.04% in GGBS compared to 19.9–24.9% in OPC) and higher amount of CaO (33.7–43.84% in GGBS compared to 62.1%). Al_2_O_3_ is also higher in GGBS compared to OPC (14.63–16.7% in GGBS and 4.95% in OPC). The water cooling and granulation processes associated with the manufacturing of GGBS produces glassy amorphous material as demonstrated by scanning electron microscope (SEM) 2θ images showing a hump between 25° and 35°. The same observation was noted of anhydrous GGBS images obtained from X-ray diffractometers (XRD) showing glassy material with negligibly small amounts of crystalline material [3]. In general, GGBS is much finer (fineness greater than 350 m^2^/kg) compared OPC, which increases reactivity and strength development at early ages.

Other popular aluminusilicates include fly ash and metakoaline. In order to achieve the desired mechanical properties, fly ash as activator requires higher dosage of sodium oxide and curing at elevated temperatures compared to ground granulated blast furnace slag (GGBS) [4,5]. For the most part, GGBS can be used as sole binder in production of concrete if activated by alkaline solution of higher pH. Compared to OPC concrete, alkali-activated slag (AAS) concrete offers several advantages including high and rapid strength development, and resistance to chemical attacks [6]. Slag can be activated using various alkaline compounds such as sodium hydroxide (NaOH), sodium carbonate (Na_2_CO_3_), potassium hydroxide (KOH), sodium silicate, or combinations of these alkalis. Sodium silicate is reported to be a more effective activator in terms of strength development compared to sodium carbonate [7].

If not addressed, rapid setting of AAS concrete makes it unsuitable for construction practice. Tests conducted by Li et al. [8] indicated that using sodium carbonate (Na_2_CO_3_) to replace part of sodium silicate (at equivalent Na_2_O content) prolongs the setting times of AAS concrete, but compressive strength development was slowed down as well. However, the strength of concrete where slag was activated using Na_2_CO_3_ continued to increase beyond 28 days and the slope of the growth curve continued to increase as well [9].

When developing AAS, it is often advantageous to combine GGBS with other cementitious materials to enhance mechanical properties and durability. Partial replacement of GGBS in AAS or OPC concrete with silica fume was found to enhance compressive strength [10]. Silica fume, however, is a relatively expensive by-product of the manufacture of silicon and ferrosilicon alloys from high-purity quartz and coal in a submerged-arc electric furnace. The escaping gaseous SiO oxidizes and condenses in the form of extremely fine spherical particles of amorphous silica (SiO_2_). The motivation for using it with OPC is that silica in the form of glass (amorphous) is highly reactive, and the smallness of the particles speeds up the reaction with calcium hydroxide produced by the hydration of Portland cement. The very small particles of silica fume can enter the space between the particles of cement, and thus improve packing.

## 2. Alkali-Activators and Hydration Products of AAS

### 2.1. Alkali Activators

**Figure 1:**
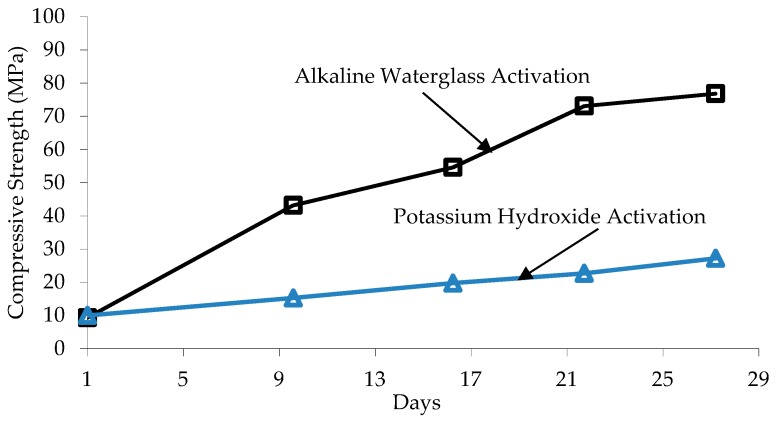
Compressive strength development of 16 mm × 16 mm × 160 mm alkali-activated slag (AAS) mortar samples activated using water glass compared to potassium hydroxide [3].

The most effective materials to activate aluminusilicates (GGBS, fly ash, etc.) include alkali hydroxide ROH, nonsilicic salts of weak acids R_2_CO_3_, R_2_S, RF, or silicic salts of the R_2_O·*n*SiO_2_, where R indicates an alkali metal ion such as Na, K, or Li [11,12]. Jin et al. [13] used MgO to activate GGBS paste and determined that the main hydration product is C-S-H in addition to a hydrotalcite-like phase that produces less porous structure and higher compressive strength. However, excessive hydrotalcite could causes cracks in the paste which could affect strength and durability [13]. Indeed, the most reported alkaline activators in the literature, include sodium hydroxide (NaOH), potassium hydroxide (KOH), sodium carbonate (Na_2_CO_3_), and sodium silicates which is also available as water glass (Na_2_O·*n*SiO_2_·*m*H_2_O). These alkaline activators produce concrete and paste with different short-term and long-term properties. Brough and Atkinson [3] reported that KOH produced more rapid reaction, less homogeneous microstructure, and lower strength compared to AAS mixes activated using sodium silicates. Compared to water glass, KOH offers high compressive strength within one day of casting, but strength development slows down significantly after one day. Figure 1 shows compressive strength development of 16 × 16 × 160 mm^3^ AAS mortar samples activated using water glass compared to samples activated using potassium hydroxide [3].

In general, the high pH of the alkaline activator promotes dissolution of GGBS, which drives chemical reaction and strength development [14]. Wang et al. [15] demonstrated that silicates activated slag produces concrete with better mechanical properties and stability compared to concrete made with slag activated using sodium hydroxide. Indeed, Shi et al. [16] demonstrated that Na_2_SiO_3_ activated GGBS produces heat of evolution that increases with decrease in water-to-binder (w/b) ratio and vice versa, unlike NaOH activated slag whose heat of hydration produced in response to w/b ratio was similar to OPC pastes.

### 2.2. Hydration Products of Alkali-Activated Slag

AAS hydration products form at the early stages, within one day, by dissolution and precipitation mechanisms, then the reaction proceeds by solid state mechanism, but regardless of the activator type the main hydration product is calcium silicate hydrate (C-S-H) with low Ca/Si ratio [6,17]. In comparison, hydration of OPC and production of needle-like C-S-H is slower, and therefore, leads to slower strength development compared to AAS concrete [17]. Other hydration products include hydrotalcite and Aluminate-Ferrite-mono-sulfate (AFm) hydrate phase having the general formula [Ca_2_(Al,Fe)(OH)_6_] X xH_2_O [17]. Water glass activated AAS for example produces poorly crystalline C-S-H even after one year. We recall that in OPC concrete, C-S-H is poorly crystalline for the most part and with variable pore size distribution [18], but morphology and chemical composition is different from C-S-H produced by AAS. When NaOH alkaline activator is used, semi-crystalline C-S-H is formed even at very early ages but when water glass is used for activation the degree of crystallinity of C-S-H is low even after one year [6]. Gifford and Gillott [7] argued that C-S-H produced by hydration of AAS is of high degree of crystallinity and lower basicity compared to C-S-H produced by hydration of OPC. AAS-based concrete should generally be more durable compared to OPC concrete. That is because the sodic reaction products associated with AAS hydration have lower solubility compared to the calcic reaction products associated with hydration of OPC [7]. However, the high alkalinity of the pore solution characteristic of AAS pastes makes concrete that uses AAS susceptible to carbonation and alkali–silicate reaction (ASR).

C-S-H produced through activation of GGBS is a foil-like phase characterized by high Si concentration and grows in pore spaces. In contract, topotactic growth of needle-like C-S-H is predominant during OPC hydration, which occurs at relatively lower silicate concentration [17].

### 2.3. Reactivity and Hydration Products of Un-Activated GGBS

Hydration of GGBS produces C-S-H even without adding alkaline activators, although at a slow rate depending on physical and chemical properties. ASTM C 989 classifies GGBS into grade 80, 100, or 120 based on the slag activity index. Amongst the chemical properties affecting hydraulic reactivity of GGBS are the basicity (CaO + MgO + Al_2_O_3_)/SiO_2_ and chemical modulus ((CaO + MgO)/SiO_2_). In addition, the presence of calcium sulfate in GGBS contributes to activation of GGBS [19]. GGBS without high basicity, modulus, and calcium sulfate showed high compressive strengths on mortar specimens and hydration products including C-S-H and ettrignite (Ca_6_Al_2_(SO_4_)_3_(OH)_12_·26H_2_O).

## 3. Mechanical Properties of Alkali-Activated Slag Concrete and Mortar

Compressive strength of AAS mortars after 28 days of curing increases with dosages of NaOH alkaline solution from 4% to 8% [20]. Aliabdo et al. [21] confirmed the same observation of increasing the compressive strength with increase of molarity of the sodium hydroxide solution in a study that used an alkaline activator solution consisting of a combination of sodium hydroxide (NaOH) and sodium sulfate (Na_2_SO_3_). However, decrease in compressive strength of AAS mortars was observed at higher dosages of alkaline solution [15]. For the same alkaline activator solution and dosage, compressive strength increases with curing temperature [22]. Nasr et al. [23] indicated that compressive strength of AAS mortars increases with in the percentage of Na_2_O of the weight of GGBS up to 8%, but then decreases with increase in percentage Na_2_O to 10% and 12%. This was the case for AAS samples under hydrothermal curing at temperatures of 25 °C as well as 200 °C. The optimum percentage of Na_2_O then shifts to 4% for hydrothermal curing under temperature 400 °C and 600 °C. The optimum Na_2_O percentage shifts for water-cared samples. Bondar et al. [24] tested AAS concrete with dosages of 4%, 6%, and 8%, and confirmed that the dosage of Na_2_O is key parameter to achieving desired workability and compressive strength, in addition to the silicate modulus and paste content.

Rapid strength development of AAS mortars from 7 to 28 days in comparison to OPC mortars is attributed to the very thin protective layer around the unhydrated GGBS grains systems. Such layer around unhydrated OPC grains is much thicker leading to slower strength development in OPC mortars [17].

**Figure 2:**
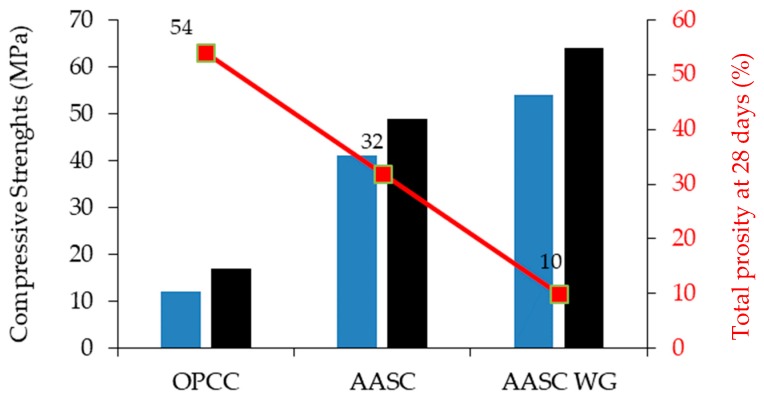
Compressive strength after 7 and 28 days of curing, and total porosity of 100 mm × 100 mm × 100 mm cubes of OPC concrete (OPCC), alkali-activated slag (AAS) activated using water glass (AAS-WG), AAS activated using NaOH (AAS-N) [25].

Puertas et al. [25] noted that AAS concrete activated using sodium silicate solution (commercial water glass) develops higher compressive strength after 7 and 28 days of curing compared to OPC concrete and compared to mixes activated used NaOH. Figure 2 shows the compressive strength development of OPC concrete (OPCC), AAS concrete activated using water glass (AAS-WG), and AAS activated using NaOH (AAS-N).

Manjunath and Narasimhan [26] studied tensile strength and compressive strength of self-consolidating concrete (SCC) with AAS as only binder and GGBS as fine and coarse aggregates. The alkaline activator solutions consisted of a combination of sodium hydroxide and water glass with Na_2_O values of 7%, 8%, and 9% by weight of slag. However, the ratio of SiO_2_/Na_2_O was kept constant at 1.0 for all mixes. Compressive strength was determined on 100 × 100 × 100 mm^3^ cubes and splitting tensile strength was measured by testing 100 mm in diameter by 200 mm in height cylindrical samples. The w/b ratios ranged from 0.33 to 0.37 and the corresponding compressive strengths of cubes after 28 days of curing ranged from 71.3 to 80.1 MPa.

Partial replacement of GGBS in AAS concrete/mortar with silica fume imparts favorable properties, especially if optimum amounts of GGBS and silica fume are selected. There is a striking similarity between the effect of silica fume on OPC concrete and the effect of AAS concrete in terms of enhancing compressive strength up an optimum percentage of silica fume. Kwon et al. [27] tested mortar samples that use slag as binder and activated using Ca(OH)_2_. Mortar sample that contained 15% silica fume developed higher compressive strength and elastic modulus compared to mortars that contained only slag after 1, 3, 7, 14, 28, 56, and 91 days of curing. The highest strength occurred after 7 days of curing with limited increase from 14 to 91 days. In experimental studies of binary (OPC–silica fume) concrete, where OPC was partially replaced with 5%, 10%, 15%, or 20% silica fume, it was found that compressive strength after 3, 7, and 28 days of curing increased with increase in silica fume quantity up to an optimum ratio of 15%, followed by a drop in compressive strength when 20% of OPC was replaced with silica fume [10]. Aydin [28] tested AAS mortars in which GGBS was partially replaced 10% or 20% of silica fume, but the author did not include the optimum 15% replacement percentage established by investigators. Compressive strength of AAS mortars with 10% silica fume was higher than mortars with 20% silica fume and higher than the control mortars made with OPC. However, the optimum replacement ratio of GGBS with silica fume does not necessarily produce the best shrinkage control characteristics in AAS, as discussed later in this paper. Compared to OPC silica fume has a much higher surface area (22,000 m^2^/kg for silica fume compared 500 m^2^/kg for OPC). This contributes to the high pozzolanic reactivity and pore filling and densification of microstructure through production of additional hydration reactions, which leads to higher compressive strength.

Rostami and Behfarnia [29] studied the effect of partial replacement of slag in AAS concrete with silica fume at percentages of 5%, 10%, and 15% by weight of slag. The alkaline activator solution consisted of a combination of sodium hydroxide (NaOH) and sodium silicate (Na_2_SiO_3_). Sodium hydroxide solution was prepared by dissolving pellets with 98% purity in water. The sodium silicate solution used has the ratio of SiO_2_/Na_2_O = 2.35 (SiO_2_ = % 36.5, Na_2_O = % 15.5, H_2_O = % 48). The authors noted that 28-day compressive strength increased increasing the percentage replacement of slag with silica fume from 5% to 15%.

## 4. Effect of Rheology, Workability, Flowability, and Mixing Time on Mechanical Properties of AAS Concrete and Mortar

Puertas et al. [25] studied the effect of AAS activator type on the rheology of concrete mix. Two activator solutions were examined, NaOH solution and sodium silicate solution (water glass). Rheology of concrete is adversely affected by longer mixing time when the binder used is OPC or slag activated with NaOH. However, longer mixing time improved both the rheology and mechanical properties of concrete when the binder is slag activated using water glass (WG). This is consistent with the finding of Palacios et al. [30]. Compared to control mixes prepared using OPC, AAS mixes had larger slump regardless of the activator type used. Specimens tested for compressive strength were 100 mm cubes demolded after 24 h of casting and cured for 7 days and 28 days. 

Degree of fineness of GGBS affects the flow rate of AAS mortars. The higher the degree of fineness the slower the flow rate but the higher the 28-day compressive strength [1]. The enhanced compressive strength associated with increased fineness of GGBS is because of the decreased porosity, increased surface density, and higher amount of hydrated GGBS particles. The specific surface area of AAS pastes was reported to be 25% higher than OPC pastes [31,32]. Therefore, it is necessary to balance the higher fineness of GGBS that is needed to enhance mechanical properties with the workability and flowability needs of the construction project. The effect of slag fineness on increasing compressive strength is not limited to 100% slag based concrete. Amin et al. [33] demonstrated that mortar strength increased with increase in fineness of electric arc slag (EAS) when used to partially replace OPC at percentages from 10% to 30%.

The dosage of Na_2_O and activator modulus (SiO_2_/Na_2_O) affects the microstructure and mechanical properties of AAS concrete. Al-Otaibi [34] noted that when the activator modulus is SiO_2/_Na_2_O = 1.0, increasing the dosage of Na_2_O decreases the porosity, while at modulus of 1.65, increasing the dosage of Na_2_O increases the porosity.

**Figure 3:**
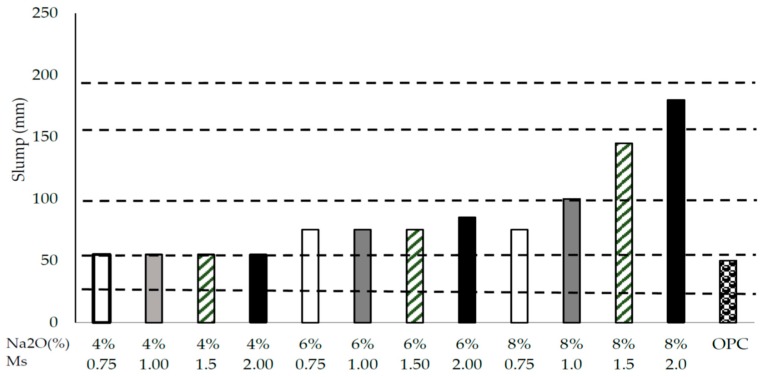
Slump of AAS with different dosages of Na_2_O and silicate moduli [24].

The dosage of Na_2_O and silicate modulus of the activator solution also affect the flowability of AAS. As shown in Figure 3, for a specific silicate modulus, the higher the dosage of sodium oxide the higher the slump of AAS mix. This increase in slump is more predominant in the case of silicate modulus, as high as 2.0, where a sodium oxide dosage of 8% produces much higher slump compared to all tested dosages and compared to the equivalent control OPC [24]. The higher the dosage of sodium oxide the higher the slump of AAS.

In general, flowability of self-consolidating concrete that contains large amounts of GGBS and fly ash is affected by aggregate density. Valizadeh et al. [35] demonstrated that the slump flow diameter decreases as the percentage of heavy weight aggregates in SCC mix increases.

The relatively faster setting time of AAS concrete activated using water glass is a shortcoming that may hamper its commercial utilization compared to OPC concrete. However, Palacios et al. [30] demonstrated that extending the mixing time can also extend the initial set time of water glass activated AAS concrete by nearly three hours. Naphthalene-based high-range water reducing admixtures (HRWR) that are commonly used with SCC was shown to enhance fluidity in AAS activated using NaOH and extend setting time as well. Initial and final setting times of AAS concrete more sensitive to curing temperature compared to OPC. Experiments by Ya-min et al. [22] indicate that at 7 °C the initial setting time of AAS mortar is longer than similar OPC mortar, while at 30 °C the initial setting time of AAS mortar becomes much shorter than similar OPC.

## 5. Effect of Curing Methods, and Exposure to Elevated Temperature on Mechanical Properties

El-Hassan et al. [36] studied the performance and microstructure of AAS concrete subjected to different curing regimes for durations up to 28 days. Three mixes were prepared to examine the effect of air curing, the second set of three mixes is for the effect of intermittent water curing, and third set is for the effect of continuous curing. Intermittent water curing mimics the construction practice of water curing for 7 days, then exposure to air. AAS concrete samples submerged under water continuously develops higher compressive strength up to the age of 7 days compared to air-curing and intermittent water curing, but by the age of 28 days, samples subjected to intermittent curing developed higher compressive strength compared to air-curing and continuous water submerging methods. Nasr et al. [23] reported that hydrothermal curing of AAS pastes under temperature from 25 °C to 800 °C yields the highest compressive strength compared to water curing as well as curing under ambient conditions. Furthermore, increasing the dosage of Na_2_O from 2% by weight of GGBS increases the compressive strength up to an optimum value that depends on the curing method and curing temperature, then further increase in Na_2_O decreases the compressive strength. For example, for hydrothermal curing the optimum value of Na_2_O is 8% for curing temperatures of 25 and 200 °C. However, the optimum value of Na_2_O for hydrothermal curing is 4% when the curing temperatures are 400 °C and 600 °C.

In order to study the effect of alkaline content on durability and mechanical properties, it is common to look at the percentage of Na_2_O by weight of GGBS. The typical range of Na_2_O examined is 4–10% by weight of GGBS. Up to curing temperature as high as 800 °C, increase in Na_2_O from 4% to 6% is accompanied by significant increase in compressive strength [37]. However, unlike curing at elevated temperature, Guerrieri and Sanjayan [38] reported that oven drying of AAS paste at only 40–50 °C leads to total disintegration and loss of strength of the samples. The investigators attributed loss of strength and sample disintegration to loss of chemically bound water which lead to the disintegration of the C-S-H gel. This effect of oven drying occurred on samples activated using commercial sodium silicate liquid combined with sodium hydroxide (NaOH).

The short setting time that adversely affect the utilization of AAS activated using certain alkaline activators such as water glass is attributed to fast formation of C-H-S caused by binding at early age of Ca^2+^ ions available in GGBS to the silicates available in water glass [30]. Silane coupling agents (SC) are proposed as retarders that can extend setting time of AAS slurries used in deep water oil well cementing applications [2]. Curing temperature was shown to affect strength development and setting time of AAS concrete [39]. Water-to-binder (w/b) ratio is also an important factor as setting time of AAS paste was shorter at w/b of 0.4 compared to higher w/b of 0.5 [31,40].

Curing of AAS concrete at temperature between 7 °C to 15 °C delays setting time to match concrete made with OPC [22]. Curing at temperature between 7 °C to 15 °C also delays shrinkage of AAS concrete along with associated cracking, reduces strength at early ages, but does not affect long-term compressive strength [22]. For example, the compact microstructure of AAS concrete after 28 days of curing at 30 °C, will take 90 days if the same concrete is cured at 7 °C. Conversely, curing at higher temperature produces AAS paste that is more compact, with lower porosity, and finer pore size distribution. Similarly, Mohamed and Najm [41] demonstrated that air curing at relatively higher temperature (45 °C) than ambient (after 3 days of submerging in water) produces higher 28-day cube strength, compared to cubes cured under water at ambient lab temperature of 22 °C. Standard cubes cured by restraining moisture from escaping using membrane-forming chemical compound, produced lower 28-day compressive strength than samples cured under air at higher temperature (45 °C).

Rostami and Behfarnia [29] noted that water curing of AAS concrete samples produces higher compressive strength after 90 days of curing compared to samples cured under plastic cover. The same observation applies to samples tested after 28 days of curing but the increase in strength was smaller. The results were consistent for AAS samples in which slag was partially replaced with 5%, 10%, and 15% silica fume. AAS concrete samples tested were 100 mm × 100 mm ×100 mm cubes prepared using w/b ratio of 0.47 and compressive strength increased with increase in silica fume replacement percentage. The activator solution was prepared using a mixture of sodium hydroxide (NaOH) and sodium silicates Na_2_SiO_3_. Sodium silicate solution consisted of SiO_2_ = % 36.5, Na_2_O = % 15.5, H_2_O = % 48 (Na_2_O/SiO_2_ = 2.35). Sodium hydroxide solution was prepared by dissolving solid NaOH to form 4 M solution. The ratio of sodium hydroxide to sodium silicate was 3.

Although moderate temperature increase contributes to faster hydration reaction, very high temperature from 200 to 800 °C decreases tensile strength compared to control unheated samples. Tests by Behfarnia and Shahbaz [42] showed that AAS samples exposed to 200 °C loss up 10% of the tensile strength while samples subjected to 800 °C loss 80% to 90% of its original tensile strength. AAS samples subjected to temperature of 300 °C or higher experienced significant loss of compressive strength compared to control OPC mortars. Pan et al. [43] demonstrated that AAS samples lost nearly 65% of the original compressive strength at ambient temperature when heated to 600 °C. Aslani and Asif [44] demonstrated that for heavy weight SCC, residual compressive strength increases with increase in temperature, but drops at elevated temperatures of 600 °C and 900 °C. 

**Figure 4:**
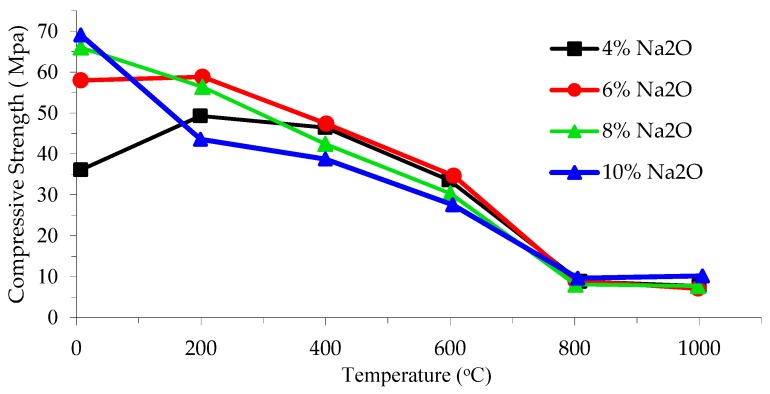
Effect of elevated temperature and sodium oxide concentration on residual compressive strength [37].

Tran and Kwon [37] studied the effect of sodium oxide concentration (Na_2_O) of the alkaline activator solution on residual compressive strength of 40 mm × 40 mm ×160 mm AAS mortar samples. Figure 4 shows that compressive strength loss begins or continues at 200 °C but significant loss of compressive strength occurs when mortar AAS samples are subjected to more than 600 °C, regardless of the content of Na_2_O.

**Figure 5:**
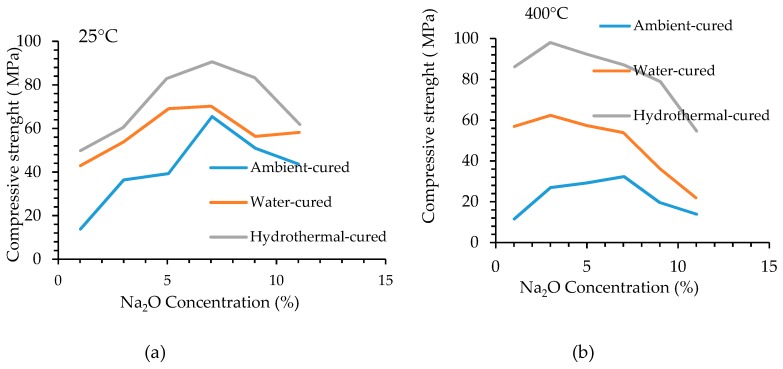
Effect of Na_2_O dosage (%) on compressive strength at (**a**) 25 °C, (**b**) 400 °C [23].

It is recognized that for a specific silicate modulus, there is an optimum sodium oxide dosage that produces the highest compressive strength. Tests by Nasr et al. [23] indicate that optimum dosage depends on temperature. Figure 5 shows that the optimum dosage of sodium oxide for maximum compressive strength at 25 °C is different from optimum dosage at 400 °C [23].

## 6. Durability—Alkali–Silica Reaction

In ordinary Portland cement (OPC) mortar and concrete, the alkali–silica reaction (ASR) takes place between potentially reactive aggregates and the alkalis present in cements (Na_2_O + K_2_O), Ca(OH)_2_ under favorable humidity conditions. ASR may cause concrete expansion and cracking of concrete along with significant reduction in compressive strength in the long term [40]. The high alkali content in AAS concrete leads to higher expansion due ASR compared to OPC concrete [45].

In experimental investigations, borosilicate glass was used to partially or fully or replace sand and assess the potential of AAS mortars for ASR. The absence of one of these factors reduces or can even inhibit reaction and, consequently, expansion. Equivalent alkalis in OPC are usually under 0.8%, but equivalent alkalis in AAS pastes may exceed 3%, thereby increasing the potential ASR expansion. In OPC, ASR occurs due to the presence of alkalis in the pore solution that will react with reactive silica in aggregates. Equation (1) was proposed by Plum and Poulsen [46] to describe the process of forming the expansive gel.

Ca(OH)_2_ + SiO_2_ + M_2_O + H_2_O → *n*_1_Na_2_O·*n*_2_CaO·*n*_3_SiO_2_·*n*_4_H_2_O (1)

where M stands for sodium (Na) or potassium (K).

For the reaction to proceed in OPC pastes, the presence of free calcium ions (${\mathrm{Ca}}^{2++}$) must be made available through portlandite (Ca(OH)_2_). Therefore, the absence of portlandite (Ca(OH)_2_) in AAS pastes reduces the potential for ASR. However, the high concentration of alkalis in AAS pastes remains a concern as they can still react with reactive silica in aggregates.

ASTM C1260-14 [47] is commonly used to determine the potential alkali reactivity of aggregates. The test is conducted by exposed mortar samples to NaOH solution, therefore, the alkali content of the cement is not a significant factor in determining the reactivity of the aggregates.

**Figure 6:**
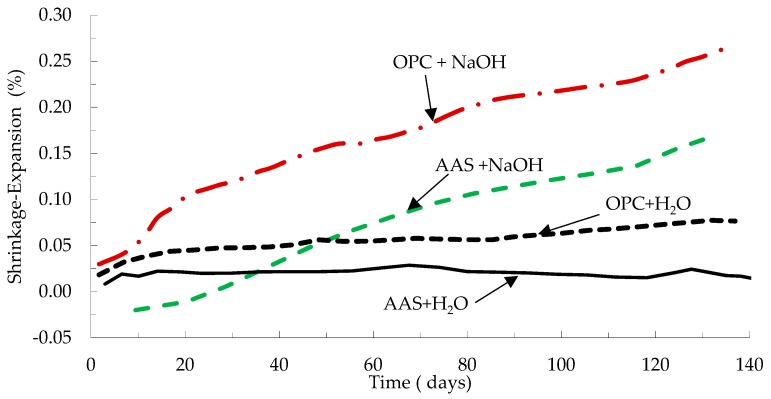
Expansion due to alkali–silica reaction of 25 mm × 25 mm × 230 mortar samples. Expansion AAS + NaOH = AAS mortars stored in 1 N NaOH solution; AAS + H_2_O = mortars stored in de-ionized water; OPC + NaOH = OPC mortars stored in 1 N NaOH solution; OPC + H_2_O = OPC mortars stored in deionized water [48].

Fernandez-Jimenez and Puertas [48] studied Alkali–aggregate reaction in mortar mixes using AAS as binder and compared the results to OPC samples. The aggregate used in making the samples contained 21% reactive silica. The mixes were prepared using slag activated with calcium hydroxide NaOH solution with Na_2_O equals to 4% by weight of slag. The activator solution to slag ratio was 0.57. They concluded that ASR expansion occurs in AAS specimens but at a slower rate compared to OPC mortar specimens. The authors observed expansion of samples due to the formation of sodium and calcium silicate hydrate reaction products with rosette-type morphology. After 140 days, AAS mortar specimens immersed in water showed very little expansion compared to OPC samples immersed in water. As shown in Figure 6, AAS mortar samples showed less expansion compared to OPC mortar samples immersed in NaOH solution. Shi et al. [20] investigated alkali–silica reaction (ASR) of mortars prepared using slag activated using 4%, 6%, and 8% wt Na_2_O by mass of slag. The accelerated ASR test was conducted by submerging mortar samples in 1 mol/L of NaOH solution and ASR expansion of mortar samples was measured up to 28 days. Before adding the NaOH solution, the alkalinity of the pore solution was higher in AAS mortars compared to Portland cement mortars. However, 28 days after adding the NaOH solution, the alkalinity of the pore solution of the mortars was higher in Portland cement mortars than in AAS mortars. As a result, ASR expansion was lower in AAS mortars compared to the Portland cement mortars.

Gifford and Gillott [7] concluded that AAS concrete is less susceptible to deleterious expansion due to ASR reaction compared to OPC concrete. The authors used 6% Na_2_O by weight of slag to activate slag. They observed higher rate of consumption of reactive silica during the early days of the reaction. However, during the early weeks of curing, they observed an excess of large voids and cavities which accommodated the silica gel produced by ASR reaction and resulted in reduced expansion.

## 7. Durability—Shrinkage, Weight Loss, and Pore Size Distribution in AAS

Four types of shrinkage are generally recognized to occur in concrete: (1) plastic shrinkage, (2) autogenous shrinkage, (3) drying shrinkage, and (4) carbonation shrinkage. Plastic shrinkage occurs if the top surface of the element dries before concrete sets. If surface drying occurs faster than the rate that bleeding water migrates to the top, shrinkage cracking is likely to occur. Autogenous shrinkage in OPC concrete occurs due to cement hydration and may occur due to the combined effect of reduction of volume of hydrated system compared to the volume of the original water and cement, and self-dessication caused by consumption of water during the hydration process. In OPC concrete, autogenous shrinkage occurs during the first 24 h, most dominantly in high strength concrete where w/b ratio is less than 0.4. However, in AAS concrete, autogenous shrinkage was shown to continue until 91 days [27]. This is due to the continuation of slag reaction and consumption of moisture in AAS concrete for a longer time compared to OPC concrete [27]. Autogenous shrinkage is relatively small for most OPC concrete mixes compared to drying and plastic shrinkage. Adding 15% silica fume to replace AAS decreases autogenous shrinkage [27]. High shrinkage of concrete increases the risk of cracking, which provides opportunities for aggressive substances to penetrate concrete and could accelerate corrosion of steel reinforcing bars [49].

It is convenient to classify pore sizes within a concrete system into micropores, mesopores, and macropores, using the International Union of Pure and Applied Chemistry (IUPAC) system for classification of pore sizes, shown in Table 1. 

The capillary pores comprise both the mesopores and macropores, and together they constitute the water filled space existing between original cement grains [50]. Micropores constitute part of the calcium silicate hydrate gel component. Shrinkage under practical conditions depends on loss of water from the mesopores and also the size of the macropores, which determines how easily water may be lost from the mesopores. In the case of alkali-activated slag, the proportion of pores in the micropore size range tend to be higher than OPC.

Collins and Sanjayan [50] conducted analysis of the incremental pore size distribution data and showed that AAS concrete has a much higher proportion of pore sizes within the mesopore range compared to OPC concrete for sample cured for 3, 7, 28, and 56 days. As shown in Table 2, the proportion of pores within the mesopore classification ranges from 74.0% to 82.0% for AAS concrete compared with 24.7–36.4% for OPC concrete [50]. The higher percentage of mesopores is the reason behind the high drying shrinkage of AAS concrete compared to the equivalent OPC concrete [50]. Similarly, analysis of weight loss data of AAS paste shows that the loss of drying water occurs from mesopores is higher than the loss of drying water from the already smaller amount of mesopores in OPC paste.

Zhu et al. [51] demonstrated that adding calcium hydroxide (Ca(OH)_2_) to AAS concrete decreases the amount of mesopores along with autogenous and drying shrinkage. However, calcium hydroxide increases plastic shrinkage as it accelerates hydration reaction during the first 120 h.

Experimental studies by Collins and Sanjayan [50] shows that drying shrinkage of Zhu et al. [51] studied the effect of adding Ca(OH)_2_ to AAS concrete to assess the effect on shrinkage characteristics. The authors investigated plastic shrinkage, autogenous shrinkage, and drying shrinkage. Five and 10% of the slag content was replaced with Ca(OH)_2_ which resulted in increased plastic shrinkage but reduced autogenous and drying shrinkage. Ca(OH)_2_ increased hydration at early age, up to 120 h, which contributed to increased plastic shrinkage. Mesopores, which are typically linked to autogenous and drying shrinkage, decreased by adding Ca(OH)_2_ to mixes that contained water-to-binder ratio (w/b) of 0.45. Decreased mesopores (2 to 50 nm) contributed to reduction in autogenous and drying shrinkage. The Ca/Si ratio in the C-S-H gel of samples containing Ca(OH)_2_ increased to 1.4 in 28 days, which the authors believed contributed to decrease of autogenous and drying shrinkage.

Collins and Sanjayan [50], studied pore size distribution and drying shrinkage in AAS paste in comparison to equivalent OPC paste samples. The activator solution was prepared from powdered sodium metasilicates and hydrated lime (Ca(OH)_2_). They concluded that drying shrinkage is much higher in AAS paste compared to equivalent OPC paste. This higher drying shrinkage is associated with higher percentage of mesopores (2 to 50 nm) in AAS paste compared to OPC. The pores in the macro range are significantly fewer in AAS paste compared to OPC paste, therefore, drying of water from mesopores affects AAS more than OPC paste. The higher drying shrinkage in AAS pastes is due for the most part to the capillary tensile forces developing during drying, more than the loss of moisture. The parameter r_s_, defined as the radius of pores where the meniscus form, such that the pores whose radii are smaller than r_s_ are assumed to be filled with liquid water while pores larger than this are dry [52]. As the drying progresses, the parameter r_s_ would decrease. It is assumed by researchers that the smaller the parameter r_s_ the larger the capillary tensile forces set up at the meniscus (the interface between water and air), hence the higher the resulting shrinkage [52]. Ye and Radlinska [53] attributed the high drying shrinkage in AAS concrete compared to OPC concrete to the reorganization and rearrangement of calcium-alumina-silicate-hydrate (C-A-S-H) layers under capillary stress. The structural incorporation of catios in C-A-S-H layers reduces its stacking regularity and makes it easier to collapse and redistribute during drying. High temperature curing was suggested to decrease shrinkage in AAS concrete by strengthening/improving the coalescence/bonding between adjacent C-A-S-H nanoparticles [54].

**Figure 7:**
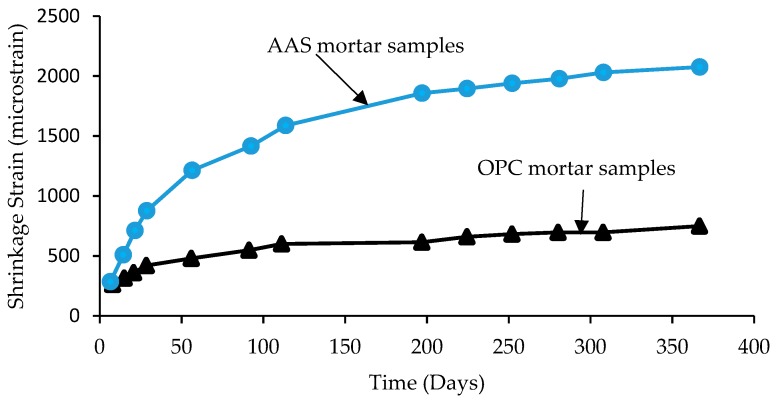
Shrinkage of 75 × 75 × 285 mm AAS samples compared to OPC samples [50].

Generally speaking, weight loss during drying is higher in OPC concrete compared to AAS concrete samples, yet drying shrinkage is higher in AAS samples compared to OPC samples. Therefore, drying shrinkage is not entirely due to weight loss of weight from concrete [50]. Figure 7 shows that shrinkage strain in AAS concrete samples is consistently higher than OPC concrete samples. However, after 365 days, the difference between drying shrinkage of AAS and OPC becomes constant [50].

Mortars prepared using GGBS activated with Ca(OH)_2_ exhibited reduced drying and autogenous shrinkage when cured at elevated temperature of 60 °C for 91 days compared to mortar samples cured at 20 °C. Similarly, when 15% of GGBS is replaced with silica fume and curated at elevated temperature, shrinkage with decreased even further compared to samples without silica fume [27].

**Figure 8:**
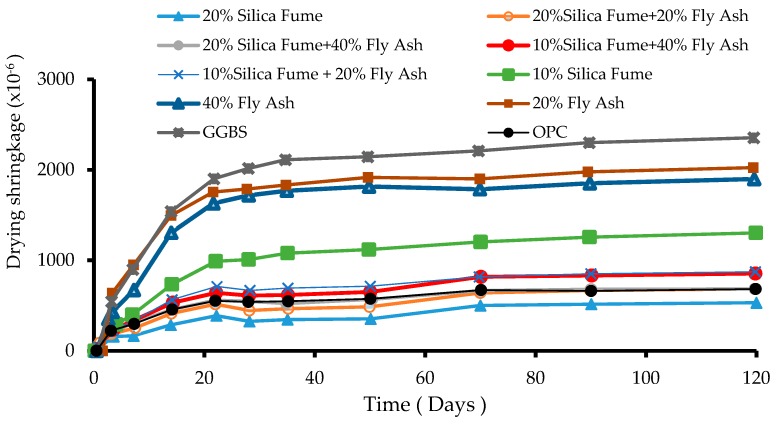
Drying shrinkage of 25 × 25 × 285 mm^3^ AAS concrete samples compared to OPC. Shrinkage of AAS samples where GGBS (ground granulated blast furnace slag) is partially replaced with various percentages of silica fume (SF) and fly ash (FA) [28].

Drying shrinkage in AAS concrete may be reduced through partial replacement of GGBS with silica fume [28]. Partial replacement of GGBS by 10% or 20% silica fume was more effective in reducing drying shrinkage in AAS mortars compared to replacing GGBS with 20% or 40% fly ash. However, replacement of GGBS in AAS mortar with 20% silica fume produces slightly lower compressive strength compared to 10% replacement percentage [28]. It may be concluded that the significant reduction in drying shrinkage of AAS with 20% silica fume, to even below control OPC control, may outweigh the relatively smaller compressive strength in AAS mortars containing 10% silica fume. As shown in Figure 8, partial replacement of GGBS in AAS concrete with 20% silica + 20% fly ash is highly effective in reducing the overall shrinkage of AAS concrete.

Aydin [28] also found that the drying shrinkage of certain ternary AAS mixes is as low as that of OPC mortars, such as 10% silica fume + 20% fly ash, 10% silica fume + 40% fly ash, 20% silica fume + 20% fly ash, 20% silica, and 20% fume + 40% fly ash. However, such ternary mixes produce lower compressive strength compared to AAS in which 10% of GGBS was replaced with silica fume. Through optimization and desirability studies, the optimum AAS mix contained 60% GGBS + 20% fly ash + 20% silica fume, in terms of high compressive strength and drying shrinkage combinations [28].

Bílek et al. [55] demonstrated that a dosage of 1% shrinkage reducing admixture (SRA) was effective in decreasing drying shrinkage of AAS mortars by 80% for samples water cured for 4 days, then exposed to drying. However, AAS mortar samples with the same SRA dosage did not show much improvement in drying shrinkage when the curing period was 28 days. This was attributed to leaching of SRA from mortar samples during prolonged water curing. Mortars containing SRA exhibited up to 30% lower 28-day compressive strength compared to control mortars that did not [55].

## 8. Durability—Sulfate, Acid, and Chloride Attack

In binary OPC + GGBS concrete, the resistance to chloride penetration increases with increase in GGBS content. Concrete in which 80% of OPC was replaced with GGBS experienced very low passing charge in chloride penetration test, even after one day of curing [56].

Rostami and Behfarnia [29] noted that resistance to chloride penetration increased when AAS concrete mixes were partially replaced with 5%, 10%, and 15% silica fume. The highest resistance to chloride penetration was exhibited with AAS samples where concrete is replaced with 15% silica fume. Similarly, the permeability of AAS concreate samples decreases as the percentage of silica fume replaced slag increased from 5% to 15%. AAS mix was activated with a combination of sodium hydroxide solution and sodium silicate solution. The ratio of sodium hydroxide solution to sodium silicate solution was 3 to 1 and the total alkaline solution to slag ratio was 0.45.

Komljenovic et al. [57] concluded that AAS concrete exhibits better resistance to sulfate (under 5% Na_2_SO_4_ solution) attack compared concrete with a binder of blend of OPC + slag. The enhanced resistance to sulfates is attributed to the lack of reactants such as portlandite (calcium hydroxide), and the fact that aluminum is mainly present in C-A-S-H and hydrotalcite gels. Similarly, AAS mortar developed higher strength and demonstrated higher when exposed to acetic acid compared to control OPC samples of the same grade [58].

## 9. Durability—Carbonation of Alkali-Activated Slag Concrete

Carbonation begins when CO_2_ in the atmosphere diffuses into the pore structure of concrete which contains moisture and together they form carbonic acid. Carbonic acid then reacts more readily with calcium hydroxide, but also with calcium silicate hydrate, calcium aluminate hydrate and ettrignite. The result is the formation of various forms of calcium carbonate [59]. Carbonation of the pore solution in concrete and mortar results in reduction of alkalinity which leads to removal of the protective *passivity* layer of oxide around reinforcing steel. Once the protective oxide layer is compromised, susceptibility of reinforcing steel bars to corrosion increases. Reduction in alkalinity associated with carbonation is believed to be the leading cause of decalcification in calcium aluminate hydrates such as C-(A)-S-H phase, katoite (C_3_AH_6_), and strätlingite (C_2_ASH_8_) formed in alkali-activated slag mortars or concretes [59]. Therefore, unlike in OPC concrete, carbonation of alkali-activated slag paste occurs directly in the C-A-S-H gel because of the lack of portlandite (Ca(OH)_2_), leaving an alumina-containing remnant siliceous gel in addition to CaCO_3_. When subjected to the same carbonation environment, concrete pastes made of the popular alkaline solution consisting of NaOH and water glass experienced higher carbonation levels compared to similar AAS paste samples activated using NaOH alone [59]. This resulted in higher loss of compressive strength in AAS paste samples activated using NaOH/water glass compared to AAS paste activated using NaOH.

The carbonation depth in AAS concrete increases with increase of temperature (from 10 °C to 30 °C) and relative humidity (RH), up to relative RH of 70% when the test was conducted at CO_2_ concentration of 20% [60]. As carbonation age increases, pore volume of AAS paste increases, but average pore diameter decreases. In addition, as carbonation age increases, the volume of carbonation products, which are calcium carbonate variants, such as aragonite and vaterite increases [60,61].

Ye and Radlinska [54] studied volume changes in mortars prepared using AAS activated using different activators. Mortar samples tested for carbonation were prepared from four alkali activator types, namely, sodium hydroxide (NaOH), sodium chloride (NaCl), sodium carbonate (Na_2_CO_3_), and potassium hydroxide (KOH). The mortar samples were subject to either atmospheric conditions or nitrogen environment. The investigators noted that AAS samples undergo expansion and disintegration under atmospheric conditions. Potassium ions (K^++^) can enter the interlayer space of calcium-alumina-silicate-hydrate (C-A-S-H) and make AAS vulnerable to carbonation. The authors argue that the high alkali content in AAS systems contribute to poor volumetric stability associated with carbonation.

**Figure 9:**
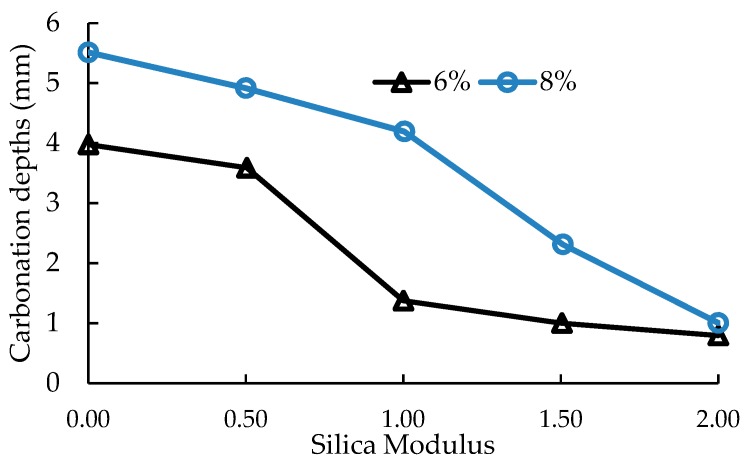
Effect of alkali dosage (6 and 8 wt% Na_2_O) and silicate modulus (Ms from 0 to 2) of alkali activators on carbonation depths measured after 7 days of CO_2_ exposure [62].

Shi et al. [62] studied carbonation of AAS mortar samples prepared used AAS activated using 6 wt.% and 8 wt.% Na_2_O by mass of slag. Samples prepared with AAS activated with alkaline solutions having different moduli were stored in carbonation chamber for 56 days. They noted that alkali-activated slag mortars are more susceptible to carbonation under accelerated carbonation condition compared to the Portland cement mortar. However, it may exhibit higher carbonation resistance under natural conditions, especially when AAS is activated with water glass having high modulus. AAS mortars with high alkali content and high silicate modulus have reduced total porosity and average pore size. This results in increased carbonation resistance and compressive strength. Figure 9 shows that increasing the silica modulus decreases the carbonation depth of AAS mortars. In addition, the carbonation depth of AAS mortars made with 8% Na_2_O is lower than AAS mortars made with 6% Na_2_O.

**Figure 10:**
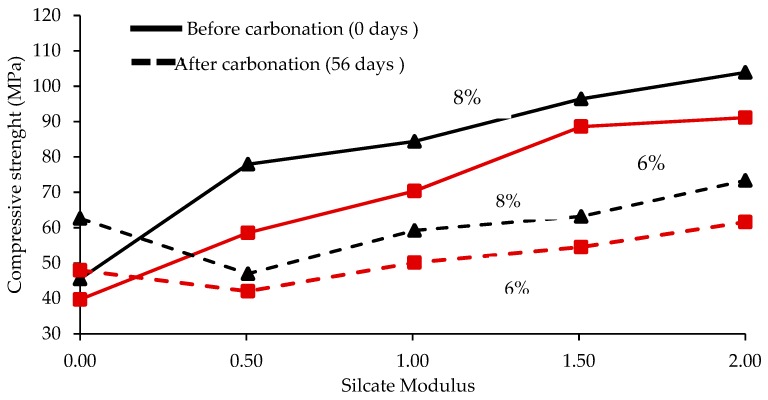
Effect of silicate modulus on compressive strength of the AAS mortars with different alkali dosages before carbonation (after steam curing) and after 56 days of carbonation [56,62].

Carbonation of the C-(A)-S-H phase leads to reduction in the molar volume of solids, which leads to increase in total porosity and average pore size, and subsequently reduction in compressive strength. However, carbonation of the C_3_AH_6_ phase leads to increase of the molar volume of solids, reduces total porosity and average pore size of the NaOH activated slag mortars, and contributes to increase of compressive strength as well. In fact, decrease in porosity of AAS concrete was shown to increase compressive strength, tensile strength, and modulus of elasticity for mixes of various molarities of NaOH (from 10 M to 14 M) in alkaline activator solution consisting of NaOH + Na_2_SO_3_ [21]. Figure 10 shows that increase in silicate modulus or alkali dosage (6% to 8%) increases the overall compressive strength of AAS mortar samples. However, for the same dosage of alkaline activator and silicate modulus, the net effect of carbonation is a reduction in compressive strength.

**Figure 11:**
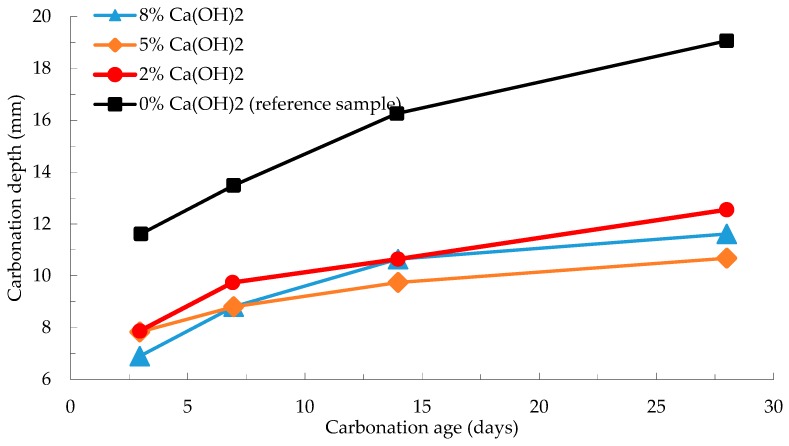
Effect of Ca(OH)_2_ dosage on carbonation depth of 100 × 100 × 100/300 mm^3^ AAS paste compared to reference AAS sample without Ca(OH)_2_ [61].

He et al. [61] reported that Ca(OH)_2_ and CaSO_4_·2H_2_O each decreased carbonation depth in AAS concrete. Figure 11 shows the effect of Ca(OH)_2_ on carbonation depth after various ages of carbonation. When 5% Ca(OH)_2_ is mixed with AAS concrete, carbonation depths at carbonation age of 3, 7, 14, and 28 days are reduced by 33%, 36%, 41%, and 45% respectively. Similarly, when 2% CaSO_4_·2H_2_O is mixed with AAS concrete, a reduction rate of carbonation depth are 25%, 14%, 24%, and 35%, respectively. The investigators used industrial water glass as activator with the solution modulus adjusted to 1.2 by adding NaOH solution. When Ca(OH)_2_ is added to AAS concrete, it will bind CO_2_ and form CaCO_3_ to decrease decalcification of the C-A-S-H gel.

It is important to note that currently implemented carbonation testing methods are accelerated under elevated CO_2_ levels. This is done in order to make the naturally slow carbonation process observable in the laboratory. These accelerated tests are extremely aggressively and unlikely represent realistic service conditions. As a result, current accelerated carbonation tests may overestimate the risk of carbonation under realistic service conditions [63]. Furthermore, under natural CO_2_ exposure conditions, the excess alkalis present in the pore solution of an alkali-activated binder may potentially maintain the internal pH at a level which is sufficiently high to protect steel in a passive state.

## 10. Durability—Freezing and Thawing

Cyclic freezing and thawing of concrete could induce serious damage, especially in the case of prolonged exposure. It was shown that concrete can lose up to 45% of its compressive strength after 50 years of exposure to freezing and thawing [64]. Interconnected capillary pores in concrete are responsible for permeability of hardened cement paste and its reduced resistance to freezing and thawing damage. Shahrajabian and Behfarnia [65] demonstrated that adding 1% to 3% nano-silica to AAS concrete improve resistance to freeze–thaw damage. Nano-silica also increased compressive strength with higher improvement for 1% dosage of nano-silica and smaller improvement for the 3% dosage. However, Fu et al. [66] conducted a freeze–thaw test on AAS concrete activated using combination sodium hydroxide and water glass, and concluded that AAS concrete possess excellent resistance to freeze–thaw damage.

## 11. Conclusions

Replacement of ordinary Portland cement with ground granulated blast furnace slag offers significant environmental benefits in terms of CO_2_ emissions and industrial production energy. The following conclusions are made regarding strength and durability of alkali-activated slag concrete and mortar:

- The main hydration product of alkali-activated slag is C-S-H in addition to (1) the hydrotalcite-like phase that produces less porous structure and higher compressive strength, and (2) the aluminate-ferrite-mono (sulphate) (AFm) hydrate phase. The high pH of the alkaline activator promotes dissolution of GGBS which drives chemical reaction and strength development.
- Compressive strength of concrete/mortar prepared using alkali-activated slag increases with increase in molarity of the alkaline activator solution. Similarly, compressive strength increases with increase in the content of sodium oxide (Na_2_O) as a percentage of the weight of GGBS up to an optimum value that depends on curing temperature, then decreases with further increase in the percentage of Na_2_O. The most commonly studied sodium oxide contents include 4%, 6%, 8%, 10%, and 12%.
- The effect of Na_2_O on compressive strength also depends on the silicate modulus of the activator solution SiO_2_/Na_2_O.
- The higher the degree of fineness of GGBS used in alkali-activated slag concrete/mortar, the higher the 28-day compressive strength. However, high degree of fineness decreases the flowability of fresh concrete.
- The lower the curing temperature, the lower the compressive strength in the short term. However, the long-term compressive is not adversely affected by low curing temperature. Similarly, water curing of alkali-activated slag concrete produces higher compressive strength compared to curing under plastic cover.
- Compared to OPC concrete, alkali-activated slag concrete demonstrates generally better resistance to chloride penetration, sulfate attack, freeze–thaw cycles, and alkali–aggregate reaction.
- Autogenous shrinkage is higher in alkali-activated slag concrete compared to OPC concrete, and continues for a longer time. Replacement up to 15% of GGBS in alkali-activated slag with silica fume decreases autogenous shrinkage. Some studies reported that drying shrinkage is also higher in alkali-activated slag concrete compared to OPC concrete, largely due to the higher percentage of mesopores in alkali-activated concrete.
- Carbonation of alkali-activated concrete/mortar is typically higher than OPC-based concrete/mortar and may lead to decalcification of C-S-H and subsequent loss in compressive strength. Carbonation in alkali-activated concrete is also affected by temperature and type of alkaline activator used.

## Figures and Tables

**Table 1:** Pore size classification.

Pore Description	Radius (nm)
Micropores	<1.25
Mesopores	1.25–25
Macropores	25–5000
Entrained air voids, entrapped air voids, preexisting microcracks	5000–50,000

**Table 2:** Pore size proportions on 75 × 75 × 285 mm concrete prisms made of AAS compared to OPC prisms [45,50].

% Mesopores			% Macropores	
Age	AAS	OPC	AAS	OPC
3	74.0	36.4	16.6	56.7
7	76.0	35.2	14.9	59.6
28	82.0	32.7	10.4	62.2
56	81.3	24.7	12.5	69

## Abbreviations

AAS	Alkali-activated slag
AFm	Aluminate-Ferrite-mono-sulfate
ASR	Alkali-silica reaction
ASTM	American Society for Testing and Material
C-A-S-H	Calcium aluminate silicate hydrate
C-S-H	Calcium silicate hydrate
GGBS	Ground granulated blast furnace slag
HRWR	High-range water reducing admixture
IUPAC	International Union of Pure and Applied Chemistry
OPC	Ordinary Portland cement
RH	Relative humidity
SCC	Self-consolidating concrete
SEM	Scanning electron microscope
SRA	Shrinkage reducing admixture
XRD	X-ray diffractometers
WG	water glass (Na_2_O·*n*SiO_2_·*m*H_2_O)

## References

[1] Yang K.H., Song J.K. (2009). Workability Loss and Compressive Strength Development of Cementless Mortars Activated by Combination of Sodium Silicate and Sodium Hydroxide. J. Mater. Civ. Eng..

[2] Jiapei D., Yuhuan B., Xuechao C., Zhonghou S., Baojiang S. (2018). Utilization of alkali-activated slag based composite in deepwater oil well Cementing. Constr. Build. Mater..

[3] Brough A.R., Atkinson A. (2002). Sodium silicate-based, alkali-activated slag mortars Part I. Strength, hydration and microstructure. Cem. Concr. Res..

[4] Junaid M.T., Kayali O., Khennane A., Black J. (2015). A mix design procedure for low calcium alkali activated fly ash-based concretes. Constr. Build. Mater..

[5] Palomo A., Grutzeck M., Blanco M. (1999). Alkali-activated fly ashes: A cement for the future. Cem. Concr. Res..

[6] Wang S.D., Scrivener K.L. (1995). Hydration products of Alkali Activated slag cement. Cem. Concr. Res..

[7] Gifford P., Gillott J. (1996). Alkali-Silica Reaction (ASR) and Alkali-Sarbonate Reaction (ACR) in activated blast furnace slag cement (ABFSC) concrete. Cem. Concr. Res..

[8] Li N., Shi C., Zhang Z., Zhu D., Hwang H.-J., Zhu H.Y., Sun T. (2018). A mixture proportioning method for the development of performance-based alkali-activated slag-based concrete. Cem. Concr. Compos..

[9] Bernal S.A., Nicolas R.S., Deventer J.S.J.V., Provis J.L. (2016). Alkali-activated slag cements produced with a blended sodium carbonate/sodium silicate activator. Adv. Cem. Res..

[10] Mohamed O.A., Najm O.F. (2017). Compressive strength and stability of sustainable self-consolidating concrete containing fly ash, silica fume, and GGBS. Front. Struct. Civ. Eng..

[11] Hardjito D., Wallah S.E., Sumajouw D.M.J., Rangan B.V. (2004). On the development of fly ash-based geopolymer concrete. ACI Mater. J..

[12] Kovalchuk G., Fernandez-Jimenez A., Palomo A. (2007). Alkaliactivated fly ash: Effect of thermal curing conditions on mechanical and microstructural development Part II. Fuel.

[13] Jin F., Gu K., Abdollahzadeh A., Al-Tabbaa A. (2015). Effects of Different Reactive MgOs on the Hydration of MgO-Activated GGBS Paste. J. Mater. Civ. Eng..

[14] Criado M., Walkley B., Ke X., Provis J.L., Bernal S.A. (2018). Slag and Activator Chemistry Control the Reaction Kinetics of Sodium Metasilicate-Activated Slag Cements. Sustainability.

[15] Wang S.D., Scrivener K.L., Pratt P.L. (1994). Factors affecting the strength of alkali-activated slag. Cem. Concr. Res..

[16] Shi C., Krivenko D., Roy P. (2003). Alkali Activated Cements and Concretes.

[17] Gruskovnjak A., Lothenbach B., Holzer L., Figi R., Winnefeld F. (2006). Hydration of alkali-activated slag: Comparison with ordinary Portland cement. Adv. Cem. Res..

[18] Guoqing G., Myers R.J., Qomi M.J.A., Monteiro P.J.M. (2017). Densification of the interlayer spacing governs the nanomechanical properties of calcium-silicate-hydrate. Nature.

[19] Park H., Jeong Y., Jeong J.-H., Oh J.E. (2016). Strength Development and Hydration Behavior of Self-Activation of Commercial Ground Granulated Blast-Furnace Slag Mixed with Purified Water. Materials.

[20] Shi Z., Shi C., Wan S., Ou Z. (2017). Effect of alkali dosage on alkali-silica reaction in sodium hydroxide activated slag mortars. Constr. Build. Mater..

[21] Aliabdo A., Elmoaty A.M., Emam M.A. (2019). Factors affecting the mechanical properties of alkali activated ground granulated blast furnace slag concrete. Constr. Build. Mater..

[22] Gu Y.-M., Fang Y.-H., You D., Gong Y.-F., Zhu C.-H. (2015). Properties and microstructure of Alkali-Activated slag cement cured at below- and about-normal temperature. Constr. Build. Mater..

[23] Nasr D., Pakshir A.H., Ghayour H. (2018). The influence of curing conditions and alkaline activator concentration on elevated temperature behavior of alkali activated slag (AAS) mortars. Constr. Build. Mater..

[24] Bondar D., Marios Q., Soutsos M., Basheer M., Provis J.L., Nanukuttan S. (2018). Alkali activated slag concretes designed for a desired slump, strength and chloride diffusivity. Constr. Build. Mater..

[25] Puertas F., Gonzalez-Fonteboa B., Gonzalez-Taboada I., Alonzo M.M., Torres-Carrasco M., Rojo G., Martínez-Abella F. (2018). Alkali-activated slag concrete: Fresh and hardened behavior. Cem. Concr. Compos..

[26] Manjunath R., Narasimhan M.C. (2018). An experimental investigation on self-compacting alkali activated slag concrete mixes. J. Build. Eng..

[27] Kwon Y.-H., Kang S.-H., Hong S.-G., Moon J. (2018). Enhancement of Material Properties of Lime-Activated Slag Mortar from Intensified Pozzolanic Reaction and Pore Filling Effect. Sustainability.

[28] Aydın S. (2013). A ternary optimisation of mineral additives of alkali activated cement mortars. Constr. Build. Mater..

[29] Rostami M., Behfarnia K. (2017). The effect of silica fume on durability of alkali activated slag concrete. Constr. Build. Mater..

[30] Palacios M., Banfill P., Puertas F. (2008). Rheology and setting of alkali-activated slag pastes and mortars: Effect of organic admixture. ACI Mater. J..

[31] Nedeljkovic M., Li Z., Ye G. (2018). Setting, Strength, and Autogenous Shrinkage of Alkali-Activated Fly Ash and Slag Pastes: Effect of Slag Content. Materials.

[32] Thomas J.J., Allen A.J., Jennings H.M. (2012). Density and water content of nanoscale solid C–S–H formed in alkali-activated slag (AAS) paste and implications for chemical shrinkage. Cem. Concr. Res..

[33] Amin M.N., Khan K., Saleem M.U., Khurram N., Niazi M.U.K. (2017). Influence of Mechanically Activated Electric Arc Furnace Slag on Compressive Strength of Mortars Incorporating Curing Moisture and Temperature Effects. Sustainability.

[34] Al-Otaibi S. (2008). Durability of concrete incorporating GGBS activated by water-glass. Constr. Build. Mater..

[35] Valizadeh A., Aslani F., Asif Z., Roso M. (2019). Development of Heavyweight Self-Compacting Concrete and Ambient-Cured Heavyweight Geopolymer Concrete Using Magnetite Aggregates. Materials.

[36] El-Hassan H., ASCE M., Shehab E., Al-Sallamin A. (2018). Influence of Different Curing Regimes on the Performance and Microstructure of Alkali-Activated Slag Concrete. J. Mater. Civ. Eng..

[37] Tran T.T., Kwon H.-M. (2018). Influence of Activator Na_2_O Concentration on Residual Strengths of Alkali-Activated Slag Mortar upon Exposure to Elevated Temperatures. Materials.

[38] Guerrieri M., Sanjayan J. (2011). Investigation of the Cause of Disintegration of Alkali-Activated Slag at Temperature Exposure of 50 °C. J. Mater. Civ. Eng..

[39] Barnett S.J., Soutsos M.N., Millard S.G., Bungey J.H. (2006). Strength development of mortars containing ground granulated blast-furnace slag: Effect of curing temperature and determination of apparent activation energies. Cem. Concr. Res..

[40] Mohamed O.A., Rens K.L., Stalnaker J.J. (2001). Time effect of Alkali-Aggregate reaction on performance of concrete. J. Mater. Civ. Eng..

[41] Mohamed O., Najm O. (2019). Effect of Curing Methods on Compressive Strength of Sustainable Self-Consolidated Concrete. IOP Conf. Ser. Mater. Sci. Eng..

[42] Behfarnia K., Shahbaz M. (2018). The effect of elevated temperature on the residual tensile strength and physical properties of the alkali-activated slag concrete. J. Build. Eng..

[43] Pan Z., Tao Z., Cao Y.F., Wuhrer R., Murphy T. (2018). Compressive strength and microstructure of alkali-activated fly ash/slag binders at high temperature. Cem. Concr. Compos..

[44] Aslani F., Asif Z. (2019). Properties of Ambient-Cured Normal and Heavyweight Geopolymer Concrete Exposed to High Temperatures. Materials.

[45] Bakharev T., Sanjayan J.G., Cheng Y.B. (2001). Resistance of alkali-activated slag concrete to alkali-aggregate reaction. Cem. Concr. Res..

[46] Plum D.W., Poulsen E. (1958). Preliminary survey of alkali reaction in concrete. Ingeniorum Int. Ed. Den..

[47] ASTM C1260-14 (2014). Standard Method for Potential Alkali Reactivity of Aggregates (Mortar-Bar Method).

[48] Fernandez-Jimenez A., Puertas F. (2002). The alkali–silica reaction in alkali-activated granulated slag mortars with reactive aggregate. Cem. Concr. Res..

[49] Wang P.M., Liu X.P. (2011). Effect of temperature on the hydration process and strength development in blends of Portland cement and activated coal gangue or fly ash. J. Zhejiang Univ. Sci. A.

[50] Collins F., Sanjayan J.G. (2000). Effect of pore size distribution on drying shrinkage of alkali-activated slag concrete. Cem. Concr. Res..

[51] Zhu X., Tang D., Yang K., Zhang Z., Li Q., Pan Q., Yang C. (2018). Effect of Ca(OH)_2_ on shrinkage characteristics and microstructures of alkali-activated slag concrete. Constr. Build. Mater..

[52] Shimomura T., Maekawa K. (1997). Analysis of the drying shrinkage behavior of concrete based on the micropore structure of concrete using a micromechanical model. Mag. Concr. Res..

[53] Ye H., Radlinska A. (2016). Shrinkage mechanisms of alkali-activated slag. Cem. Concr. Res..

[54] Ye H., Radlinska A. (2017). Carbonation-induced volume change in alkali-activated slag. Constr. Build. Mater..

[55] Bilek V., Kalina L., Novotny R., Tkacz J., Parizek L. (2016). Some Issues of Shrinkage-Reducing Admixtures Application in Alkali-Activated Slag Systems. Materials.

[56] Mohamed O. (2018). Durability and Compressive Strength of High Cement Replacement Ratio Self-Consolidating Concrete. Buildings.

[57] Komljenovic M., Bascarevic Z., Marjanovic N., Nikolic V. (2013). External sulfate attack on alkali-activated slag. Constr. Build. Mater..

[58] Bakharev T., Sanjayan J.G., Cheng Y.-B. (2003). Resistance of alkali-activated slag concrete to acid attack. Cem. Concr. Res..

[59] Li N., Farzadnia N., Shi C. (2017). Microstructural changes in alkali-activated slag mortars induced by accelerated carbonation. Cem. Concr. Res..

[60] Chen Y., Liu P., Yu Z. (2018). Effects of Environmental Factors on Concrete Carbonation Depth and Compressive Strength. Materials.

[61] He J., Gao Q., Wu Y., He J., Pu X. (2018). Study on improvement of carbonation resistance of alkali-activated slag concrete. Constr. Build. Mater..

[62] Shi Z., Shi C., Wan S., Ning W., Zhang L. (2018). Effect of alkali dosage and silicate modulus on carbonation of alkali-activated slag mortars. Cem. Concr. Res..

[63] Provis J.L., Palomo A., Shi C. (2015). Advances in understanding alkali-activated materials. Cem. Concr. Res..

[64] Mohamed O.A., Rens L., Stalnaker J. (2000). Factors affecting resistance of concrete to freezing and thawing damage. J. Mater. Civ. Eng..

[65] Shahrajabian F., Behfarnia K. (2018). The effects of nano particles on freeze and thaw resistance of alkali-activated slag concrete. Constr. Build. Mater..

[66] Fu Y., Cai L., Wu Y. (2011). Freeze–thaw cycle test and damage mechanics models of alkali-activated slag concrete. Constr. Build. Mater..

